# Supplementary material for: A large-scale analysis of bioinformatics code on GitHub
Source: PLoS One. 2018 Oct 31;13(10):e0205898. doi: 10.1371/journal.pone.0205898 (PMC6209220; doi:10.1371/journal.pone.0205898)
Supplement: S1 File — (PDF) [file pone.0205898.s001.pdf]

Supplemental information, methods and figures for

"A large-scale analysis of bioinformatics code on GitHub" by Russell et al.

Supplemental Section 1: Source code and project setup

All original code for this study is in the repository https://github.com/pamelarussell/github-bioinformatics. The repository contains a single pipeline script, src/pipeline/github_bioinformatics_pipeline.pl, that wraps all code for the study. The pipeline script is hereafter referred to as the "Perl pipeline". The Perl pipeline accepts a JSON configuration file. Pipeline steps are selected for a particular run by setting boolean values within the configuration file; this triggers other scripts in the repository to be run. A sample configuration file is provided as src/pipeline/sample_config.json. The Perl pipeline is not intended to be run from start to finish but simply to gather all computational steps in a single location for reproducibility. Throughout the Supplemental Information, pointers to source code are given by the name in the configuration file that controls the running of that code and sometimes by the main script that implements the analysis. Scripts are indicated by their relative path within the repository.

Several scripts require the list of repositories of interest, which is stored in a Google Sheet and provided as Table S4 and Table S5. See "Authentication" below for how to incorporate the sheet into a reproduction of the data and results in the paper.

Repository names

Throughout the Supplemental Information, the term “repository name” is used to refer to a GitHub username and repository separated by a forward slash, e.g., “pamelarussell/github-bioinformatics”.

Authentication

In order to reproduce the data and results in the paper using the Perl pipeline and associated code, authentication is required for Google as well as the GitHub API. Anyone wishing to reproduce the data and results in the paper needs a Google account and a GitHub account. Users will need to set up a project on BigQuery in order for dataset creation to work. The Google Sheets containing the repository lists need to be saved in the user’s Google Drive account. The Google Sheets containing the repository lists formatted for import into BigQuery also need to be saved in the Google Drive account, and BigQuery tables need to be set up that contain the data in these sheets. This can be done by first linking a BigQuery table to the sheet, then querying for the entire contents of the table and saving as a regular BigQuery table for simpler authentication. Google credentials provide access to BigQuery (where the data tables are written and stored) as well as Google Sheets (where the repository lists are stored). Google credentials are stored in a JSON key. GitHub credentials are stored as an OAuth token. For the pipeline, Google and GitHub credentials are defined in the config file, not included in the public GitHub repository for the project. R code uses the “bigrquery” package [1], which prompts for

Google login credentials and saves an OAuth token. The GitHub API is not accessed from any of the R code.

Reproducibility

All data extracted from the GitHub API, with modifications described in the next paragraphs, are available at https://doi.org/10.17605/OSF.IO/UWHX8.

Although all of the repositories studied are public on GitHub and were announced in published articles, many do not include explicit open source licenses. Therefore, the actual contents of the repositories cannot be included as a public dataset along with this paper. Instead, we have recorded the specific Git version of each file so that the exact dataset can be regenerated by other individuals. This table, provided as "file_info_main.csv" and "file_info_high_profile.csv", contains the Git URL pointing to the specific record for each file. Readers who wish to reproduce the exact dataset for the paper can copy each file info table to a BigQuery table (in a respective BigQuery project for the main or high-profile dataset) named "repos:file_info" and continue with file content extraction in the Perl pipeline by setting "generate_file_contents" to true in the config file (skipping "generate_file_info", which would create the "repos:file_info" table for current versions of repository contents).

Similarly, the GitHub Terms of Service prohibit sharing of personal identifying information including names and e-mail addresses. (We used names for the gender analysis.) We have published commit records as "commits_main.csv" and "commits_high_profile.csv" with

identifying information removed. Each published record includes an API reference for the commit so the full record can be reconstructed from the GitHub API if needed.

All of the other data extracted from the GitHub API are also included at https://doi.org/10.17605/OSF.IO/UWHX8; these can similarly be uploaded to BigQuery and the data extraction steps in the Perl pipeline can be skipped. Readers who wish to generate a dataset but do not require the exact dataset used for this paper can follow the entire Perl pipeline, which will capture the current state of repositories.

Supplemental methods

Supplemental Section 2: Identification of bioinformatics repositories on GitHub

We identified GitHub repositories containing bioinformatics code by (1) identifying published journal articles containing mentions of GitHub, (2) manually selecting bioinformatics articles from among these results, (3) automatically extracting names of GitHub repositories within the articles, and (4) manually curating the GitHub repositories using surrounding context in the articles.

Motivation for search strategy

We settled on this strategy after several previous iterations brought various issues to light. We initially considered searching GitHub itself for the term “bioinformatics”, but quickly realized that (1) the resulting set of repositories shared no common benchmark making them comparable to one another, such as having reached the point of a publication, and (2) many or most bioinformatics repositories do not explicitly mention the term “bioinformatics”. Because of these issues, we decided to use repositories that had been prepared and theoretically vetted along with peer-reviewed publications; additionally, this choice allowed us to analyze article metadata as well.

After deciding to use repositories that had been published along with papers, we attempted several automatic search strategies. A PubMed search for the term “GitHub” revealed the fact that a large proportion of the search results would not be considered “bioinformatics” articles by our community. To give a few very brief examples, non-bioinformatics topics we discovered included radiomics, pure statistics, mathematics, and articles about GitHub having no connection to biology. We decided that it would be unacceptable to include these non-bioinformatics articles. We then attempted to use machine learning to automatically classify article abstracts as bioinformatics or not, using a hand-labeled set of hundreds of abstracts. The best classifier we trained was approximately 90% accurate and still admitted sufficiently many non-bioinformatics articles to be unacceptable. We therefore decided that it would be necessary to hand-label each article as bioinformatics or not. We recognized the compromise of this decision: we would have to limit the size of the dataset to accommodate hand labeling, but believed that this would be preferable to including many irrelevant repositories that could bias the analysis.

We designed a literature search strategy (described in detail in “Literature Search” below) that would be as unbiased as possible while returning a number of results that could be reasonably hand-labeled by two members of our group. We identified 2,679 articles containing the term “GitHub” in the full text, and labeled 1,950 of these as “bioinformatics”.

We automatically extracted the names of 3,188 GitHub repositories mentioned in the 2,679 articles (described in “Automatic extraction of repository names from articles” below). Again, we quickly realized that many of these repositories should not be included in our dataset (even if the article was a bioinformatics article), the most common reason being that authors would mention repositories they had had no part in developing. (An important goal of our analysis was to analyze repositories along with the paper in which they were first published.) We therefore manually labeled each GitHub repository as being part of the work described in the paper or not.

Finally, as briefly mentioned above, part of our analysis depended on identifying the single paper that first announced each repository. Therefore, in the small number of cases where a repository was mentioned in multiple distinct papers, we used the original article announcing the repository (described in “Manual curation of repository names” below).

Table S4 lists the labels associated with each step of this decision process for each article and repository name.

Literature search

The goal of the literature search was to identify and collect articles that mention the term "GitHub" in the title, abstract or anywhere in the full text. Because the primary biomedical databases (PubMed, Embase) only contain article metadata (title, abstract, etc.) and not full text, we took a two-pronged approach. We designed a search strategy that looked for the term "GitHub" in the title/abstract, but also included computer programming terminology that could indicate the use of "GitHub" in the full text. To identify the relevant proxy language to search for, we used three approaches. First, we looked at citations that did indeed mention “GitHub” and harvested relevant associated or surrounding language. Second, we ran the search “GitHub” in PubReMiner to look for other text or MeSH terms that frequently appeared in citations that mention “GitHub”. Finally, we relied on our content knowledge to identify other relevant terms.

Because of the laborious nature of locating and processing large batches of full-text PDFs, we designed the literature search to return at most 30,000 citations. Literature searches were performed on 20 June 2017 in PubMed and Embase.com. Embase contains MEDLINE citations as well as citations unique to Embase. For both searches, the date range was set to 2008-present because GitHub was launched in 2008. No other limits were applied. The PubMed search simply looked for the term "GitHub" in all fields and yielded 1,821 results after deduplication. The Embase strategy searched the term "GitHub" in addition to other terms related to computer programming and software. This search served the dual purpose of locating the term "GitHub" in the title/abstract of Embase-only citations as well as identifying citations in both MEDLINE and Embase that had some chance of mentioning GitHub in the full text. The Embase search yielded 26,505 results after deduplication. Both searches yielded a combined total of 28,326 unique citations.

The search performed in Embase was the following:

“((algorithm:ti,ab OR toolkit:ti,ab) AND (code:ti,ab OR software:ti,ab)) OR (analysis:ti,ab AND next:ti,ab AND (framework:ti,ab OR pipeline:ti,ab OR software:ti,ab OR tool:ti,ab)) OR 'command line':ti,ab OR ((framework OR 'freely available' OR pipeline OR 'publicly available' OR workflow) NEAR/4 (code OR software)):ti,ab OR github*:ti,ab OR 'open source':ti,ab OR 'programming language':ti,ab OR (software:ti,ab AND next:ti,ab AND (application:ti,ab OR framework:ti,ab OR package:ti,ab OR pipeline:ti,ab OR program:ti,ab OR suite:ti,ab OR tool:ti,ab)) OR 'source code':ti,ab OR 'web app*':ti,ab AND [2008-2017]/py”

Due to a searcher error, not identified until after citation processing and analysis, the intended adjacency operator "next" was treated as a text word by Embase and “AND”-ed together with other search terms within the respective scope. This error did not affect the search for "GitHub" in the title/abstract. Rather it required the word "next" to appear with the associated computer/software terms. This limited overall search results, but kept the search within the manageable 30,000 citation range.

All citations were exported to EndNote X7. EndNote's full-text harvesting tool was used to batch harvest PDFs. No manual harvesting of PDFs was performed. 18,764 full-text PDFs were located by EndNote. All citations containing "GitHub" in the title/abstract and all located PDFs were exported for external programmatic analysis. We identified 2,679 articles containing the case-insensitive term “GitHub” somewhere in the full text.

Definition of bioinformatics

A detailed list of bioinformatics topic categories was compiled. First, the published scope of the journal *Bioinformatics* was downloaded on 25 June 2017 from [2]. Each category in the journal scope, along with its detailed description, was included. Second, a few additional categories were taken from the Wikipedia article on "Bioinformatics" on 25 June 2017 [3] (stable URL). Finally, an additional topic "Pipelines, wrappers, extensions, and utilities" was included to capture these software papers. Descriptions of each category taken from their sources are in Table S1.

Manual curation of bioinformatics articles

Each article identified in the literature search that contained the term "GitHub" in the full text was manually evaluated to determine if its contents pertained to bioinformatics topics. The set of articles was divided into two subsets and each subset was evaluated by one person (R.J. and P.R.) due to the large time commitment involved. For each article, the title and abstract were examined. The article was classified as "bioinformatics" if the title or abstract treated at least one of the topics in the definition of bioinformatics. The results of the manual classification are presented in Table S2.

Automatic extraction of repository names from articles

Repository names were automatically extracted from all articles identified in the literature search, including those not identified as "bioinformatics". The script was run through the Perl pipeline by setting "extract_repos_from_lit_search" to true in the config file. Briefly, the operation

of the script is as follows. The XML files of article metadata exported from EndNote were parsed and metadata for all articles were extracted. For each article, first, an attempt was made to identify repository names in the abstract by searching for and parsing matches to one of the regular expressions "github\.com/([a-zA-Z0-9_-]+/[a-zA-Z0-9_-]+)" or "([a-zA-Z0-9_-]+)\.github\.io/([a-zA-Z0-9_-]+)". If repository names were found in the abstract, these were returned and no attempt was made to analyze the full text. If no repository names were found in the abstract, the full text PDF was analyzed using the Python package pdfminer [4]; matches to the same regular expressions were identified and parsed to extract repository names. The script saved the results to a table on BigQuery and this table was saved as a Google Sheet. The table is available as Table S3.

Manual curation of repository names

Spreadsheet for manual curation of repository names.

The final curation is presented as Table S4. To create this table, the manual curation of bioinformatics articles was joined to the automatic extraction of repository names from articles to identify automatically extracted repository names contained in bioinformatics articles. A Google spreadsheet was created containing the join. From that point, this spreadsheet was manually adjusted (Table S4 contains a sheet “field_definitions” that defines the columns and explains which columns have been manually modified). The column “use_repo” in the spreadsheet contains the ultimate directive of whether the repository was to be included in the final dataset or not, and could be manually set for various reasons described below. The complete definition of the logic in this column is provided in “field_definitions”.

Manual deduplication of repository names.

Duplicate repository names were identified. Duplicates could occur when the same article was returned multiple times by the literature search, leading to multiple EndNote records. This could also happen if the same repository was mentioned in multiple different articles. In these cases, records were manually deduplicated. If the same article was returned from multiple databases, the PubMed record was kept and the other records were deleted (it was always possible to keep a PubMed record). If the same article was in the same database with two different dates, the earlier record was kept in “use_repo” and the later record was not used. In more complex cases, such as multiple distinct articles mentioning the same repository, the articles were manually examined and at most one article was set to be used in “use_repo”; the article chosen was the one originally announcing the repository.

Manual checking and correction of repository names.

For each repository name in a bioinformatics article, the surrounding context of the abstract or article was manually examined to determine if the repository contained code for the article, as opposed to the article mentioning an outside repository. This determination was manually entered in the column “repo_from_pdf_is_code_for_bioinf_paper” of the spreadsheet provided as Table S4. If a repository name had been discovered from the article abstract, only the abstract was examined during this manual process; the PDF was not examined. If a repository name had been discovered from the full text PDF, the entire PDF was examined. In some cases, errors in repository names were discovered during this manual curation process; these were manually fixed where possible. (For example, errors in repository names could be caused by ambiguously hyphenated line breaks or special formatting such as indented bullets in the

PDF, or missing spaces after the repository name in the abstract downloaded from a literature database.)

Special issues for PDFs.

In addition to the repositories automatically detected from the PDF, additional repositories not identified by the automatic process were added by searching for the string “github”; these could have been missed by the automatic script due to the issues mentioned previously. Therefore, in order for any repository names to be included from a PDF, at least one repository name needed to have been identified automatically. PDFs for which no repository names were automatically identified were not manually examined and therefore were not allowed to contribute any repository names to the final dataset.

Special situations during manual curation.

- In some cases, if multiple repositories were mentioned in an article and it was impossible to tell from context whether the repositories were developed by the article authors, we viewed the repositories on github.com to evaluate contributors to the repositories.
- Articles published in the journal *F1000* often include pointers to two code repositories: one containing stable frozen code at the time of publication and another containing the development version. In these cases, the development version was used and the stable version at the time of publication was not used, for consistency with other repositories that are all theoretically development versions.
- BioJava [5] and BioJS [6] are open source projects that collect multiple components from different contributors under a single parent GitHub repository (biojava/biojava and biojs/biojs, respectively). Components of these projects were not used because our analysis

is performed at the repository level, and components of these projects are subdirectories under a common repository.

Checking validity of repository names

After the manual curation of repository names, a script was run to verify the current existence of repositories marked to be used in the final dataset. The script was run in the Perl pipeline by setting “check_repo_existence” to true in the config file. The script prints a list of repositories whose existence could not be verified through the GitHub API. Most of these turned out to have moved or changed names; these repository names were manually corrected. This step also revealed more repository names containing errors due to the automatic parsing of the abstract or PDF; these were manually corrected. Repositories that could not be found at all were not used. After the manual modifications in this step, no issues with repository names were identified by the script.

Identification of high-profile bioinformatics repositories

In addition to the repositories identified through the literature search, we curated a set of “high-profile” projects: highly respected and well-known tools in the bioinformatics community. Most of these projects were not identified in the literature search. In many cases, high-profile projects were not hosted on GitHub at the time of publication. These projects also could have been absent from the literature search because the papers did not mention GitHub, because the papers did not match the heuristics used in the search, or because the code is not publicly available.

To avoid subjective judgements or omissions of popular tools, we chose to define high-profile projects as those generating a high volume of discussion in the leading online forum for discussion of bioinformatics topics, Biostars [7]. We accessed Biostars on 10 February 2018, compiling a list of standalone software tools that had been tagged in posts at least 100 times. We chose to draw the boundary at standalone tools because this provided a clean criterion we could use to judge the sometimes ambiguous Biostars tags, but acknowledge that our chosen criterion excludes a few popular libraries and conglomerations such as Bioconductor and Galaxy. The list included 27 tools. Through a manual web search, we were able to identify a primary GitHub repository hosting the code for 21 of these tools. Four tools do not appear to be hosted publicly on GitHub, while two tools are included under another repository already in the set of 21. In one case, Samtools [8], the project was spread across multiple GitHub repositories and we curated three repositories containing the main code for the project, bringing the number of repositories to 23. Three high-profile repositories (alexdobin/STAR, bcgsc/abyss, and chrchang/plink-ng) are also in the dataset curated from the literature search; they are included in both sets for analysis. We performed a manual search to identify the original publications describing each project; we were able to find publications for 21 of the 23 repos, while two remain unpublished. Details are presented in Table S5. This set of 23 repositories is referred to as the “high-profile” dataset, while the set identified through the literature search is referred to as the “main” dataset.

Extraction of article metadata

Metadata for articles associated with each repository were extracted from NCBI databases using the RISmed R package [9] with the script src/R/ncbi/paper_metadata_eutils.R. The script was run through the Perl pipeline separately for the main and high-profile datasets by setting “query_eutils_article_metadata” to true in the config file. Metadata retrieved include database IDs, journal information, funding information, relevant dates, article abstract, and number of citations in PubMed Central, the National Library of Medicine’s archive of open access full-text biomedical and life sciences articles.

Supplemental Section 3: Extraction of repository data from GitHub API

Several types of data were extracted from the GitHub REST API v3 [10]; each is described in a subsection below. These scripts were run separately for the main and high-profile datasets; each dataset was stored in a separate BigQuery project. The BigQuery projects and empty datasets within each project were created manually in the BigQuery web interface. Data for the high-profile dataset were extracted approximately four months after the main dataset.

Workflow components common to all data types

Python scripts were used to obtain each type of data. All scripts use the gspread library [11] to read the list of repository names from the Google Sheet containing the manual curation of repositories (Table S4). All scripts make Curl requests to the GitHub API using the PycURL library [12] and parse the JSON responses to convert information to flat records. All scripts push data to tables in Google BigQuery using the BigQuery-Python library [13].

Repository-level metrics

Repository-level metrics were extracted from the GitHub Repositories API [10] and pushed to a BigQuery table by the script src/python/gh_api_repo_metrics.py. The script was run through the Perl pipeline by setting “generate_repo_metrics” to true in the config file. Repository-level metrics include (1) repository name, (2) GitHub API URL for the repository, (3) HTML URL for the repository, (4) repository description, (5) whether the repository is a fork, (6) number of stargazers, (7) number of watchers (legacy endpoint that now returns the number of stargazers), (8) number of forks, (9) number of open issues, (10) number of subscribers (users who have subscribed to notifications; previously known as “watchers”), (11) SHA-1 hash of the most recent commit reference to the master branch, and (12) time at which the information was accessed.

File information

Information on individual files contained in each repository was extracted from the GitHub Contents API [10] and pushed to a BigQuery table by the script src/python/gh_api_file_info.py. The script was run through the Perl pipeline by setting “generate_file_info” to true in the config file. Recursive requests were constructed in order to access the entire directory structure of each repository. Information for regular files and symbolic links was retrieved. Submodules were not included because these often contain code not developed by the authors of the main repository. Information retrieved for each file includes (1) repository name, (2) file name, (3) file path, (4) file SHA-1 hash, (5) file size, (6) GitHub API URL for the file, (7) HTML URL for the file,

(8) Git URL for the file, (9) download URL for the file, (10) file type, (11) SHA-1 hash of the most recent commit reference to the master branch, and (12) time at which the information was accessed.

File creation dates

Initial commit timestamps for each file were extracted from the GitHub Repositories API [10] and pushed to a BigQuery table by the script src/python/gh_api_file_init_commit.py. The script was run through the Perl pipeline by setting “generate_file_init_commits” to true in the config file. Commits affecting each file were accessed via the repository name and path as stored in the file information table; the oldest time at which a committer committed the file was stored.

File contents

Contents of individual files were extracted from the GitHub Repositories API [10] and pushed to a BigQuery table by the script src/python/gh_api_file_contents.py. The script was run through the Perl pipeline by setting “generate_file_contents” to true in the config file. File contents were accessed via their Git URL as stored in the file information table, so that records in the two tables refer to exactly the same versions of each file. This was important due to the duration of time needed to extract all the file contents. Information retrieved for each file includes (1) repository name, (2) file name, (3) file path, (4) file SHA-1 hash, (5) Git URL for the file, (6) file contents, and (7) time at which the information was accessed. File contents were decoded from the Base64 encoding returned by the GitHub API. Files whose contents exceed 999KB in size

were included in the results table but contents were marked as “null” due to the 1MB row size limit in BigQuery and also the fact that almost none of these files contain source code.

Commits

Commit records were extracted from the GitHub Repositories API [10] and pushed to a BigQuery table by the script src/python/gh_api_commits.py. The script was run through the Perl pipeline by setting “generate_commits” to true in the config file. Attributes extracted for each commit were (1) repository name, (2) commit SHA, (3) commit API URL, (4) commit HTML URL, (5) commit comments URL, (6) commit message, (7) comment count, (8) author login, (9) author ID, (10) author name, (11) author email, (12) author commit date, (13) author API URL, (14) author HTML URL, (15) author type, (16) committer login, (17) committer ID, (18) committer name, (19) committer email, (20) committer commit date, (21) committer API URL, (22) committer HTML URL, (23) committer type, (24) SHA-1 hash of the most recent commit reference to the master branch, and (25) time at which the information was accessed. The commit author and committer may be different if the author submitted the commits via a pull request. Only commits to the default branch (usually “master”) were included.

Licenses

Repository licenses were extracted from the GitHub Repositories API [10] and pushed to a BigQuery table by the script src/python/gh_api_licenses.py. The script was run through the Perl pipeline by setting “generate_licenses” to true in the config file. For each repository, information extracted included (1) repository name, (2) license, (3) SHA-1 hash of the most recent commit

reference to the master branch, and (4) time at which the information was accessed. License information is returned by the API when it can be detected from the repository's license file. Repositories without a detectable license were recorded as "null" in the BigQuery table.

Note on iterating through files

We needed to pull down the contents of each file from our contents table in BigQuery and save it to a local file in order to analyze it with cloc (Supplemental Section 5). Although the Google Cloud API supports iterating through records in a BigQuery table, there is a limit on record size that was exceeded by many of our contents records. Therefore, we exported the contents table to multiple CSV files on Google Cloud Storage; our analysis script downloaded these CSV files locally one at a time to analyze the subset of files contained therein. Therefore, people utilizing our analysis code would need to replicate the process of saving the contents table to multiple CSV files in Google Cloud Storage.

Supplemental Section 4: Topic modeling of article abstracts

We used machine learning to infer topics for abstracts of the articles announcing each repository in the main dataset. Abstracts for the single curated article for each repository were obtained from the EndNote metadata (see Supplemental Section 2). Treating each abstract as a document, we created a latent Dirichlet allocation (LDA) model [14] using the "topicmodels" R package [15] and following the workflow in [16]. In LDA, the symbol β refers to the probability of a given term being generated from a given topic, and γ is the probability that a given document

comes from a given topic. From the LDA model, we identified terms whose β value for their top topic was at least four times larger than the second highest topic. We manually examined the top terms for each topic from this list of topic-specialized terms. We tried several values for k in the model (the number of topics) and chose $k = 8$ for further analysis due to its maximal coherence of concepts within the top terms. We manually assigned a label to each of the eight topics that captures a summary of the top terms. We classified each article abstract into one or more topics by taking all pairs of abstracts and topics with γ equal to at least 0.25. The topic modeling analysis and figures (Fig 2, Fig A, Fig B, Fig C, Fig D) were generated in paper/scripts/topics.Rmd.

Supplemental Section 5: Programming languages

We attempted to identify a programming language, count lines of code and comment, and extract comment-stripped source code for each file. The script src/python/cloc_and_strip_comments.py calls the tool cloc (version 1.72) [17] to analyze the contents of each file in each repository. The script was run through the Perl pipeline by setting "run_cloc" to true in the config file. For each file, cloc attempts to identify the programming language, number of lines of code, number of comment lines, number of blank lines, and comment-stripped source code. Files with extensions indicating they did not contain source code (e.g. ".jpg", ".pdf", ".ppt") were skipped and not run through cloc. Some files with identical contents were duplicated in the dataset, usually appearing multiple times in the same repository with different paths and/or file extensions. In cases where cloc identified different language or line counts for these duplicate files (probably due to file extension heuristics used in cloc), all

copies of the file were skipped. A similar filtering was performed on the comment-stripped code results from cloc. Results from cloc were saved to tables in BigQuery. This information was joined to other file metadata with the script src/python/run_bq_queries_analysis.py by setting “run_bq_analysis_queries” to true in the config file for the Perl pipeline.

Language execution modes were obtained from [18]. Type systems were obtained from [19], and due to the absence of the popular language R from this table, R was manually added and labeled as “dynamic” and “unsafe”. In order for the information to match the programming languages assigned to our data by cloc, in some cases language information records were copied to match the language names returned by cloc. These tables, provided as Table S6 and Table S7, were saved as Google Sheets. In order to reproduce the results in the paper, the tables must be copied to tables in BigQuery using the same procedure described in Supplemental Section 1.

Fig 3 and Fig E were generated in paper/scripts/analysis.Rmd, building on analysis performed in paper/scripts/repo_features.R.

Supplemental Section 6: Developer communities

We identified the number of commit authors and outside contributors (commit authors who are never committers) in the commit records for each repository. For commit authors, we attempted to count unique people by collapsing users with the same name or login, as individuals can contribute to a repository under multiple aliases (for example, from multiple devices with

different default name settings). For outside contributors, we counted commit authors whose author ID is never a committer ID for the repository. Counts of commit authors and outside contributors were calculated in paper/scripts/repo_features.R. The counts of forks, subscribers and stargazers were extracted directly from the GitHub API in src/python/gh_api_repo_metrics.py by setting “generate_repo_metrics” to true in the config file for the Perl pipeline.

Supplemental Section 7: Gender analysis

Inferring genders

The script src/R/gender/infer_gender.R attempts to infer a gender for each commit author, committer, and paper author in the dataset, then pushes the results to a BigQuery table. The script was run for the main and high-profile datasets through the Perl pipeline by setting “infer_gender” to true in the config file. We used the Genderize.io API [20], which is a paid service above a certain usage rate; an API key is required for the script to function. Genderize accepts a first name and optional language and country, and returns a gender call along with the estimated probability of correctness. Although many GitHub users provide their geographic location as a free-form text field and articles include academic affiliations for authors, we chose not to use this information because (1) many developers and researchers do not live in their home country, making this information potentially misleading, and (2) it is challenging to convert free-form text to uniform country codes. The result of this decision is that we lack gender calls for some ambiguous names that could possibly be resolved by adding accurate geographic

information. We note that we were only able to obtain author lists for 1,573 articles for the main dataset (covering 1,658 repositories) and and 18 articles for the high-profile dataset (covering 21 repositories) (see Extraction of article metadata), and that some author lists were not usable for gender analysis because they list first initials only. We did not use the original EndNote citations for author gender because they included first initials only.

We submitted first names (the first word before whitespace) to Genderize and accepted gender calls with a worldwide probability of 0.8 or higher. The main dataset contains 13,425 unique strings in the “author name” and “committer name” fields of the commit records and the author names of articles. Several cleaning steps reduced this to 9,286 strings that were likely to represent full names as opposed to other information such as usernames or e-mail addresses. Of these, we were able to confidently infer a gender for 7,747 unique names. Similarly, the high-profile dataset contains 1,145 unique names, 881 after cleaning, and 775 for which we were able to infer a gender. We note that, based on manual observation, there may be a slight bias against identifying genders for non-anglophone names. We also note that a few individuals appear to be in the dataset more than once with different ways of writing their name, but these are very rare. We were able to confidently infer a gender for 83.4% of cleaned names in the main dataset and 88.0% of cleaned names in the high-profile dataset.

Analysis of developer and author gender

Code for this analysis is in paper/scripts/gender.Rmd, which also created Fig 5.

Developers, commits, and paper authors by gender

For the gender breakdown of developers, we counted unique full names of authors and committers, collapsing people with the same name or login, and ignoring other identifying information such as email address. Although we could theoretically be falsely collapsing multiple individuals with the same name, we find that it is much more common for the same individual to exist in the dataset with multiple aliases. For commits, we joined commit records to genders by the full name of the commit author, and counted individual commits. For paper authors, we counted individual authorships on papers announcing the repositories.

Team composition

We analyzed team composition for the 504 projects in the main dataset for which we could infer a gender for at least 75% of developers (collapsing developers with the same name or login) and 75% of paper authors. We analyzed diversity for the 602 repositories in the main dataset for which we could infer a gender for at least 75% of developers. We defined team types as “solo female” if the team consisted of one woman, “solo male” if the team consisted of one man, “all female” if no individuals were identified as male (individuals with no gender call may actually be male), “all male” if no individuals were identified as female, “majority female” if more individuals were identified as female than male, “majority male” if more individuals were identified as male than female, and “equal” if the same number of female and male individuals were identified.

Gender diversity

We quantified gender diversity using the Shannon index [21]. The Shannon index was originally developed to quantify entropy in information theory and has been been widely used across a variety of scientific disciplines to measure diversity of categories within a set or population, including being used to quantify gender diversity in the social sciences [22,23]. We calculated

the Shannon index for gender diversity within developer teams (defined as the set of unique individuals contributing to a particular repo) and within commits (defined as the gender of the author of each individual commit to a repo, where individual authors are counted once per commit).

Supplemental Section 8: Commit dynamics

We defined project duration as the time span between the first and last commit timestamps (author commit date) for the repo at the time we extracted the data. This was accomplished by the script src/python/run_bq_queries_analysis.py. The script was run through the Perl pipeline by setting “run_bq_analysis_queries” to true in the config file. We identified the initial commit time for each file by taking the earliest timestamp of all commits touching the file; this was accomplished with the script src/python/gh_api_file_init_commit.py by setting “generate_file_init_commits” to true in the config file. Metrics describing monthly activity (mean commits per month, max consecutive months with and without commits, mean new files per month) are with respect to the number of months in the project duration. These were calculated in paper/scripts/repo_features.R. Fig 6 was created in paper/scripts/analysis.Rmd.

Supplemental Section 9: Proxy for project impact

We defined the variable “commits after publication” to be true if the latest commit timestamp at the time we accessed the data was after the day the associated article appeared in PubMed.

Articles were identified and article metadata were extracted as described in Supplemental Section 2. Repository data were extracted from the GitHub API as described in Supplemental Section 3. Fig 7 was created in paper/scripts/analysis.Rmd. The variables displayed in each panel were calculated in paper/scripts/repo_features.R.

Supplemental figures

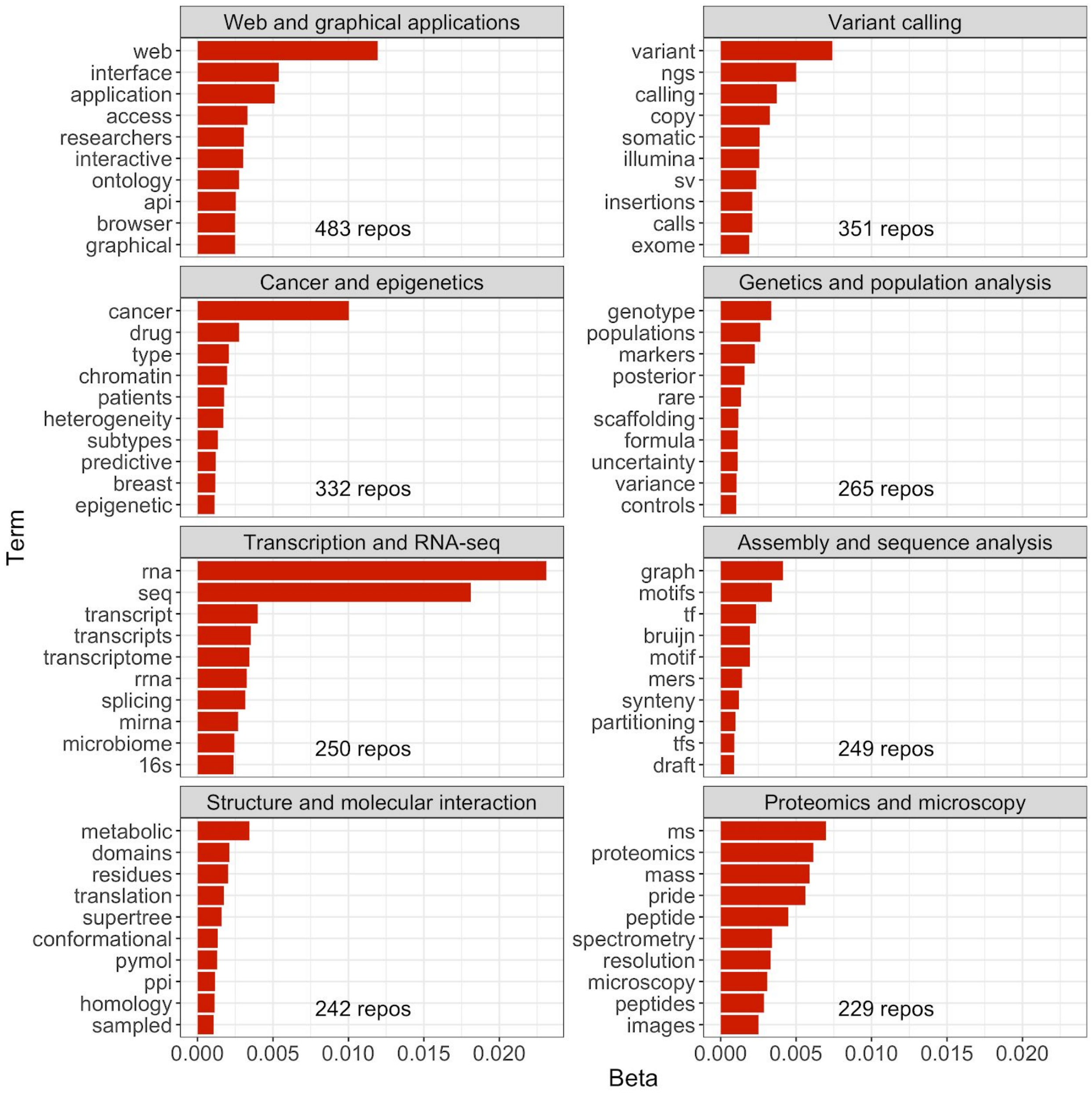

Fig A. Topic modeling of article abstracts in the main dataset. The results of a topic modeling analysis of article abstracts for the main dataset are shown. We treated each abstract as a document and created an eight-topic latent Dirichlet allocation model [14]. β represents the probability of a given term being generated from a given topic. The figure shows top terms that are sufficiently exclusive to each topic. For each topic, the listed terms have the top ten β values such that β is at least four times the β value of the second highest topic for the term. (For

example, the term “data”, which has high β values for several topics, is excluded.) The reported number of repositories for each topic is the number of articles whose abstract has a γ value (probability of coming from the topic) of at least 0.25; articles may be associated with more than one topic. The topic labels were designated manually after examining the top terms. The figure was created in paper/scripts/topics.Rmd.

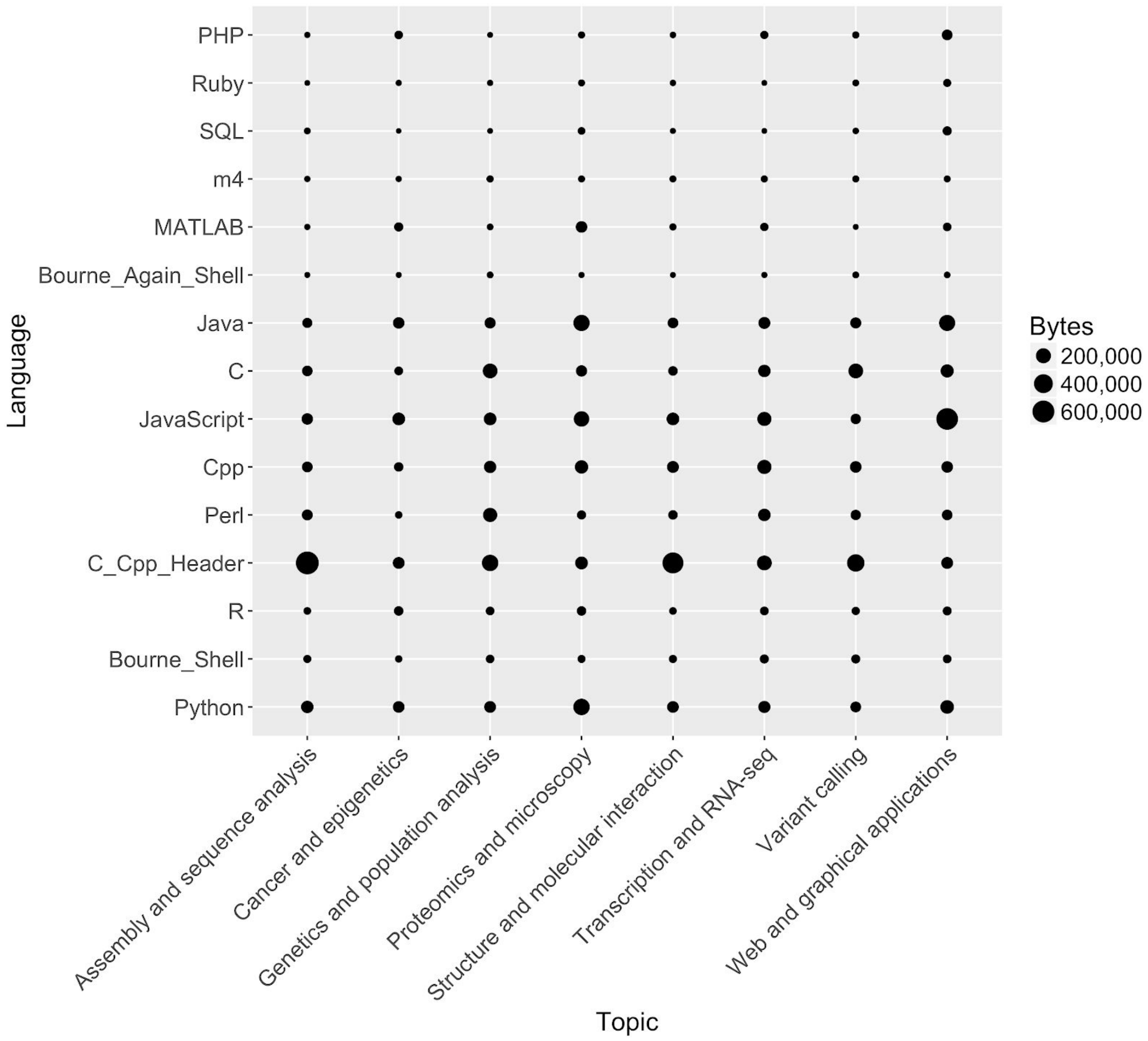

Fig B. Programming languages and article topics in the main dataset. Each repository is associated with the article that announced it. We ran topic modeling on article abstracts; see

Supplemental Section 4. The size of each dot represents the total number of bytes of code in repositories in the main dataset whose corresponding article is associated with the given topic. Only languages included in at least 50 main repositories are displayed. Articles can be associated with more than one topic; in that case, the code is counted separately for each topic. The figure was created in paper/scripts/topics.Rmd.

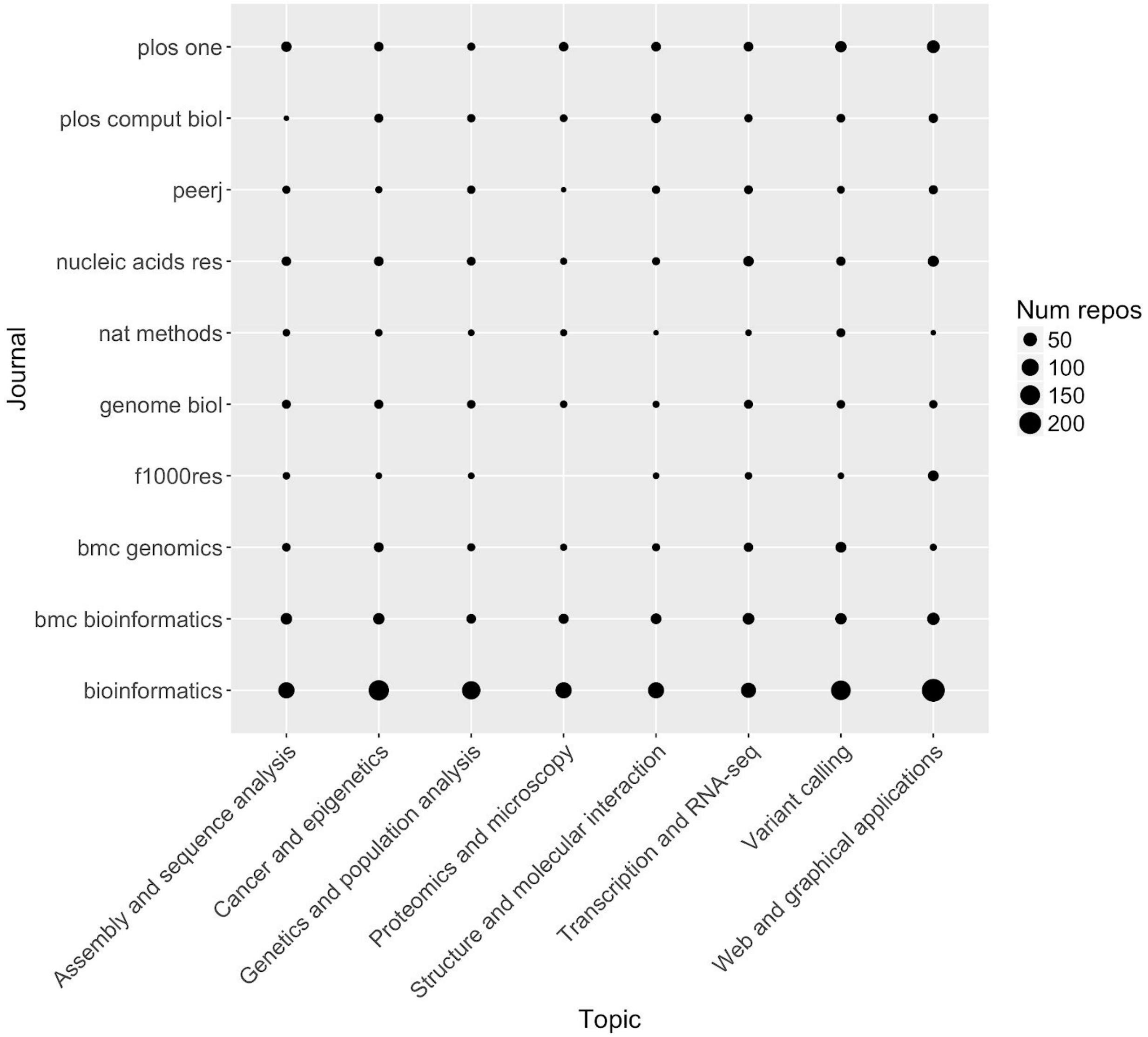

Fig C. Article topics and journals in the main dataset. The size of each dot represents the number of articles published in the given journal that are associated with the given topic. Only the ten most common journals are included. Articles can be associated with more than one topic; in that case, the journal is counted for each topic. The figure was created in paper/scripts/topics.Rmd.

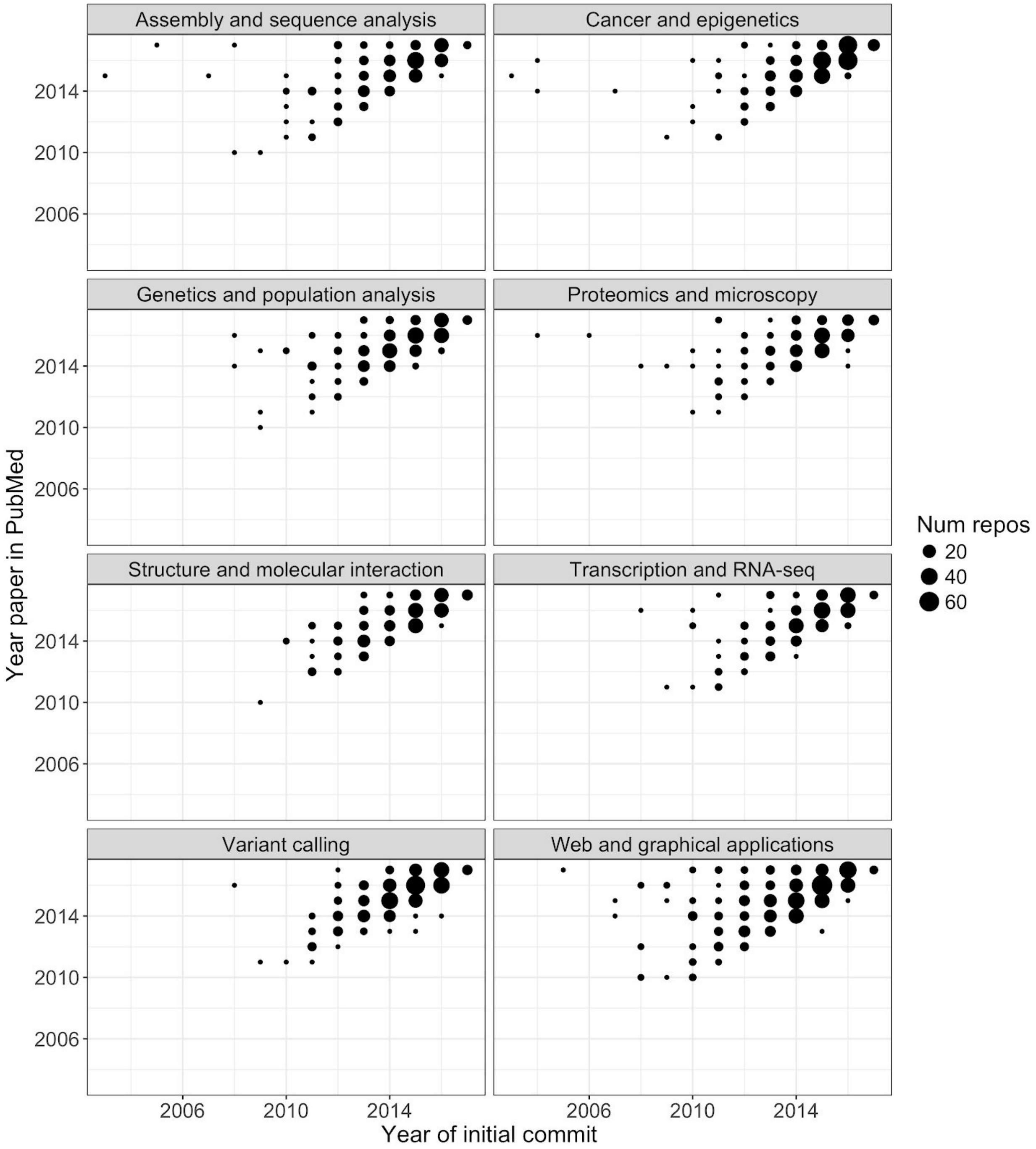

Fig D. Article topics by year in the main dataset. For each repository, the year of the first commit to the repository and the year the associated paper appeared in PubMed are shown. Articles associated with multiple topics are included in the plot for each topic. The figure was created in paper/scripts/topics.Rmd.

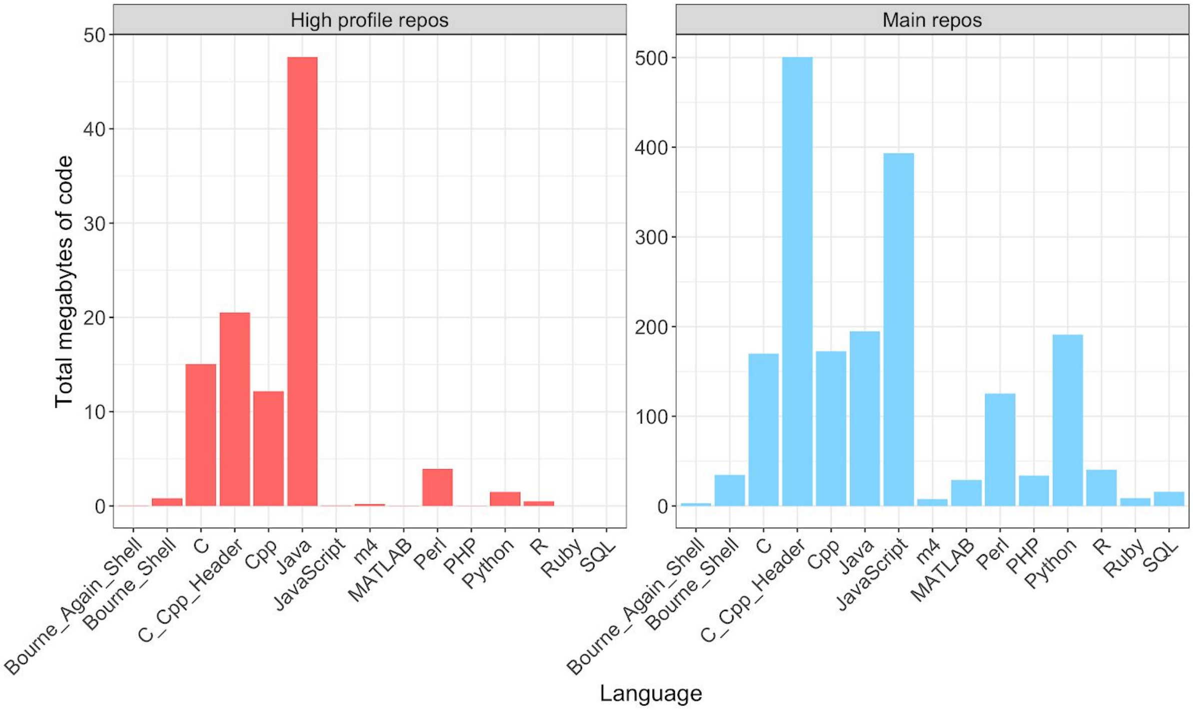

Fig E. Amount of code by programming language. The total amount of code by programming language is shown for main and high-profile repositories. The amount of code is the total size in bytes of all files identified with the language by cloc (see Methods). Only languages included in at least 50 main repositories are displayed. The large amount of code in C and C++ headers for the main repositories is largely due to two repositories that contain entire copies of the Boost C++ libraries [24] within the repo; this accounts for nearly half of the ~500Mb of code reported here. The figure was created in paper/scripts/analysis.Rmd.

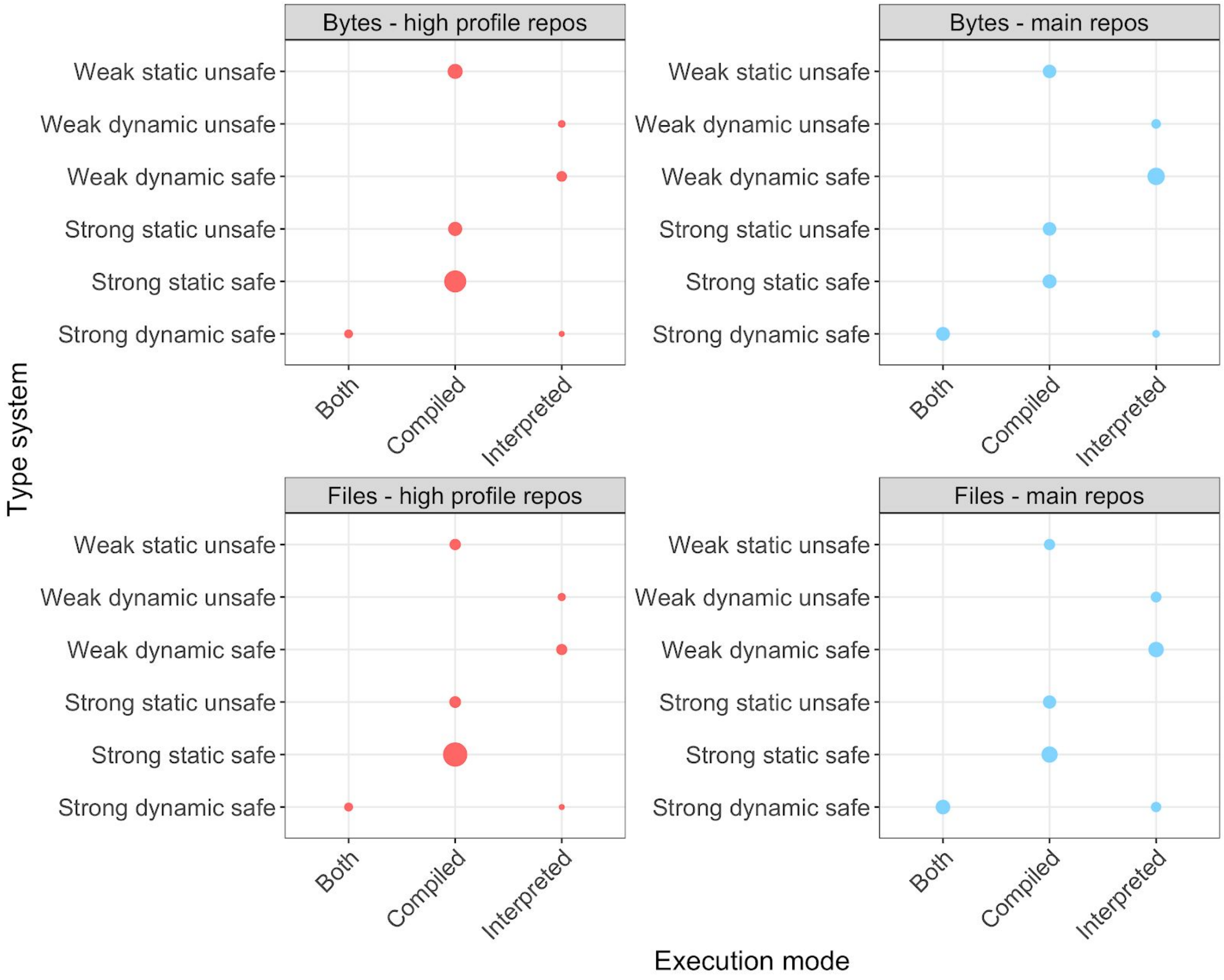

Fig F. Language features. We attempted to identify a programming language for each file in each repository. Language properties were determined for a subset of languages. The figure counts files whose language is associated with a type system and execution mode. Separate plots display total file size in bytes and total number of files. (See Supplemental Section 5.) The figure was created in paper/scripts/analysis.Rmd.

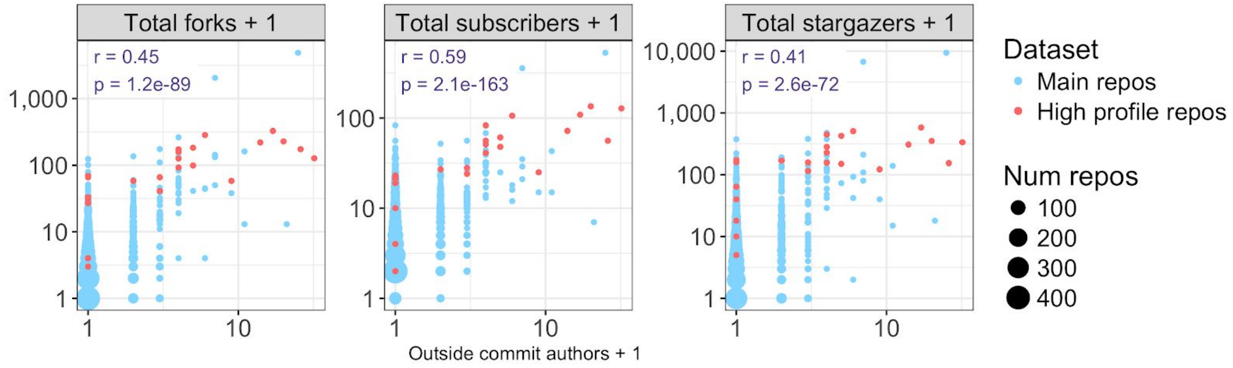

Fig G. Outside commit authors. Various measures of community engagement are plotted against the number of outside commit authors (commit authors who are never committers to the repository). Outside commit authors are determined from commit records for each repository by comparing author IDs to the full set of committer IDs for the repository. Each dot represents one repository or a set of repositories with the same values for the variables. We added one to the vertical axis variables to facilitate plotting on a log scale due to many zero values. The pearson correlation and associated p-value are displayed for each variable versus the number of outside commit authors. See Fig 2 legend for the explanation of forks, subscribers and stargazers. The figure was created in paper/scripts/analysis.Rmd.

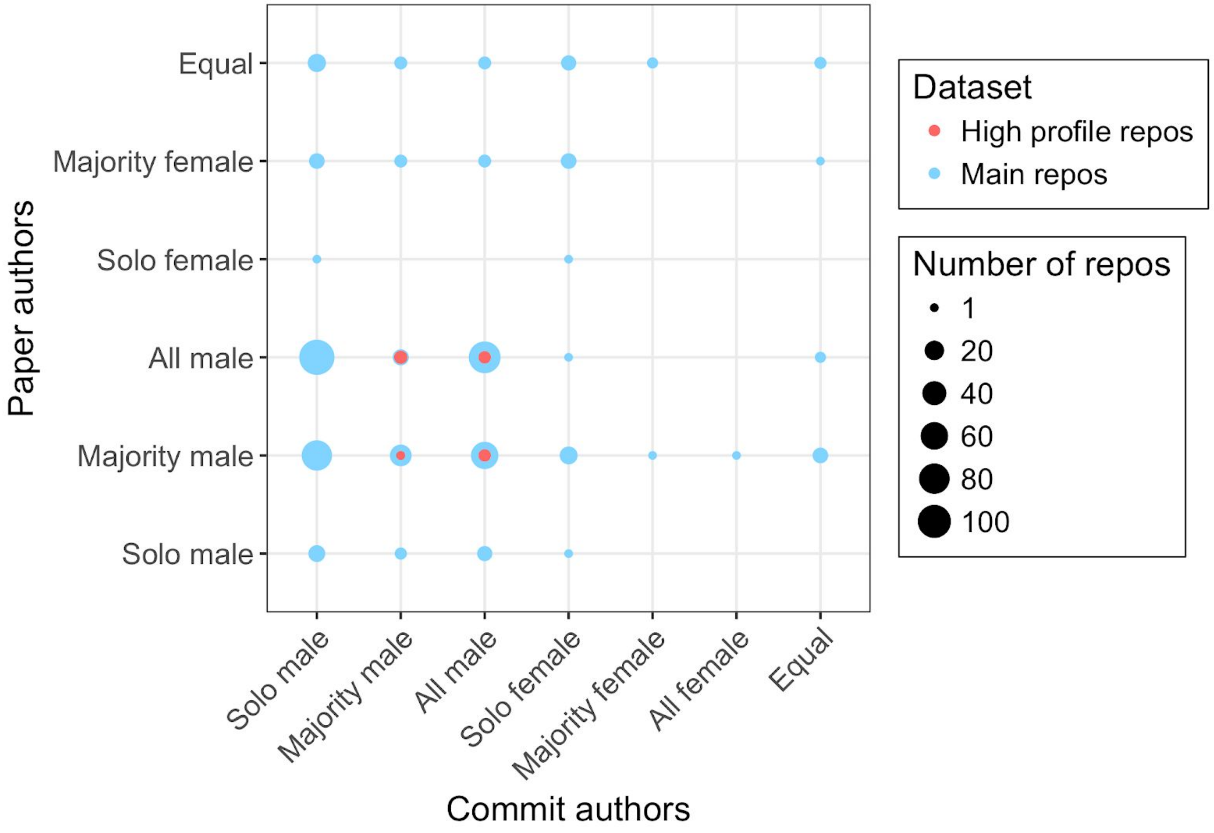

Fig H. Distribution of teams by gender composition. Developer teams are defined as the set of unique commit authors contributing to the default branch of each repository. Paper authors are the set of authors listed on the paper announcing the repository. The figure only includes 515 teams for which we could infer a gender for at least 75% of developers and 75% of paper authors. People with no inferred gender are not counted when determining categories for each team. The figure was created in paper/scripts/gender.Rmd.

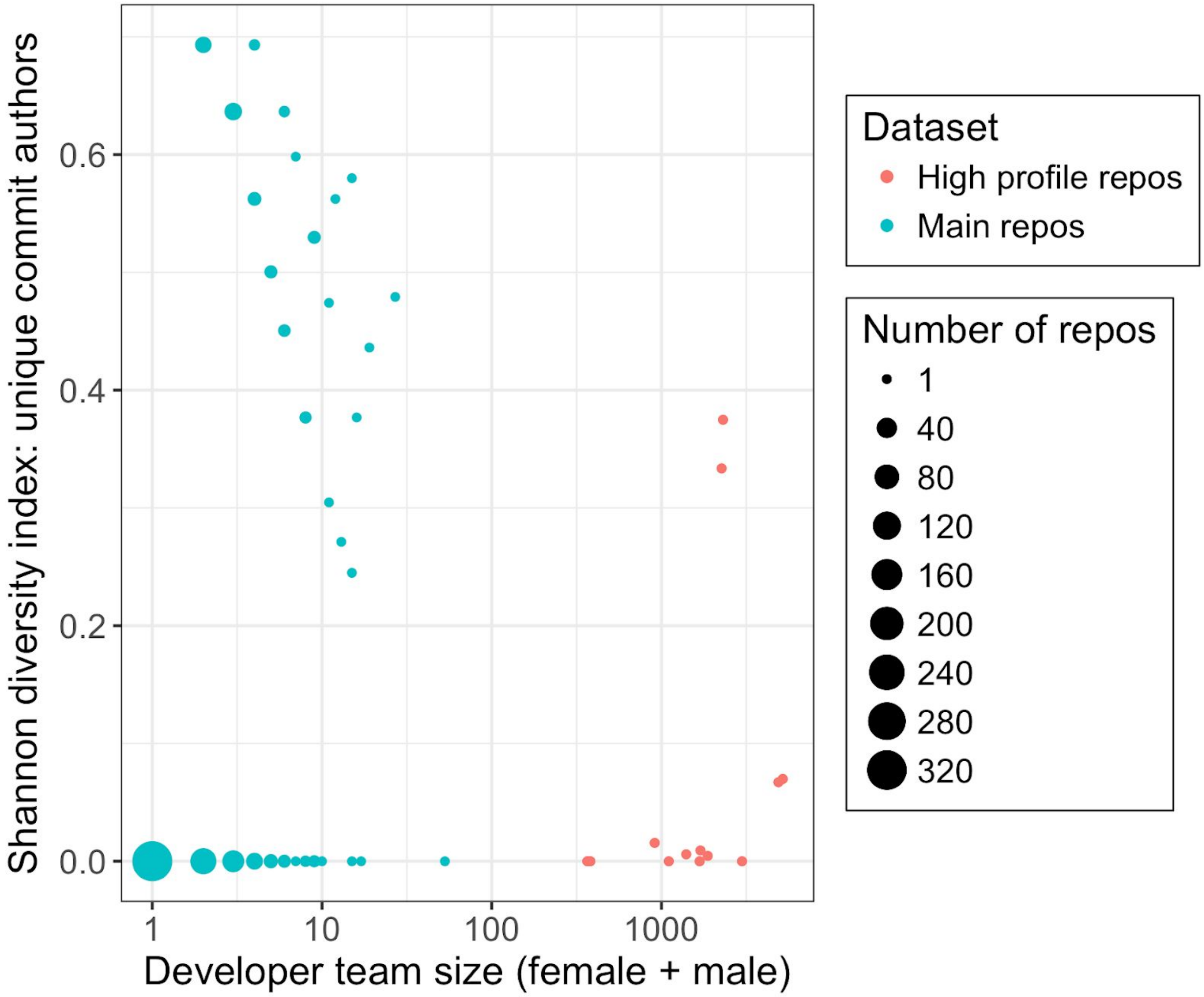

Fig I. Gender diversity and team size in the main dataset. Developer teams are defined as the set of unique commit authors contributing to the default branch of each repository. We only include 615 teams for which we could infer a gender for at least 75% of developers. The Shannon diversity index is used as a measure of gender diversity within each team. The maximum possible value of the Shannon index with two categories is ln(2) = 0.69, which is achieved for any team with equal numbers of female and male developers. Developers with no inferred gender are not counted when calculating team size or gender diversity. The figure was created in paper/scripts/gender.Rmd.

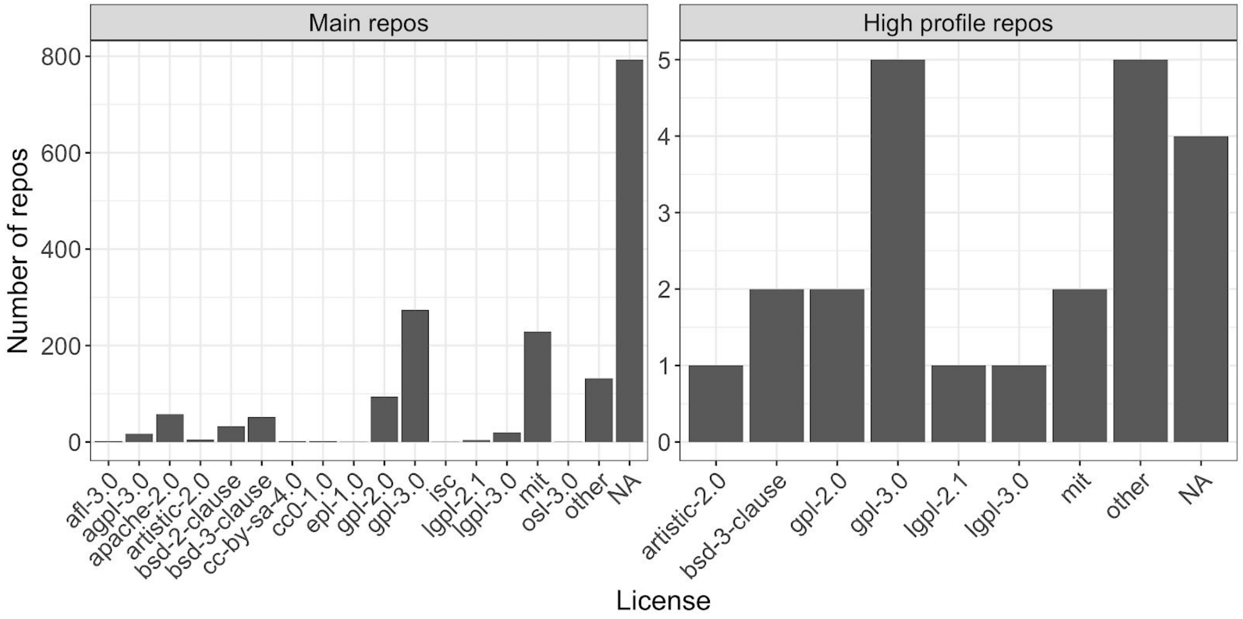

Fig J. Repository licenses. Licenses were extracted from the GitHub API, which returns a license for a repository if the license can be automatically determined from a license file. Repositories with no detectable license are counted under “NA”. The figure was created in paper/scripts/analysis.Rmd.

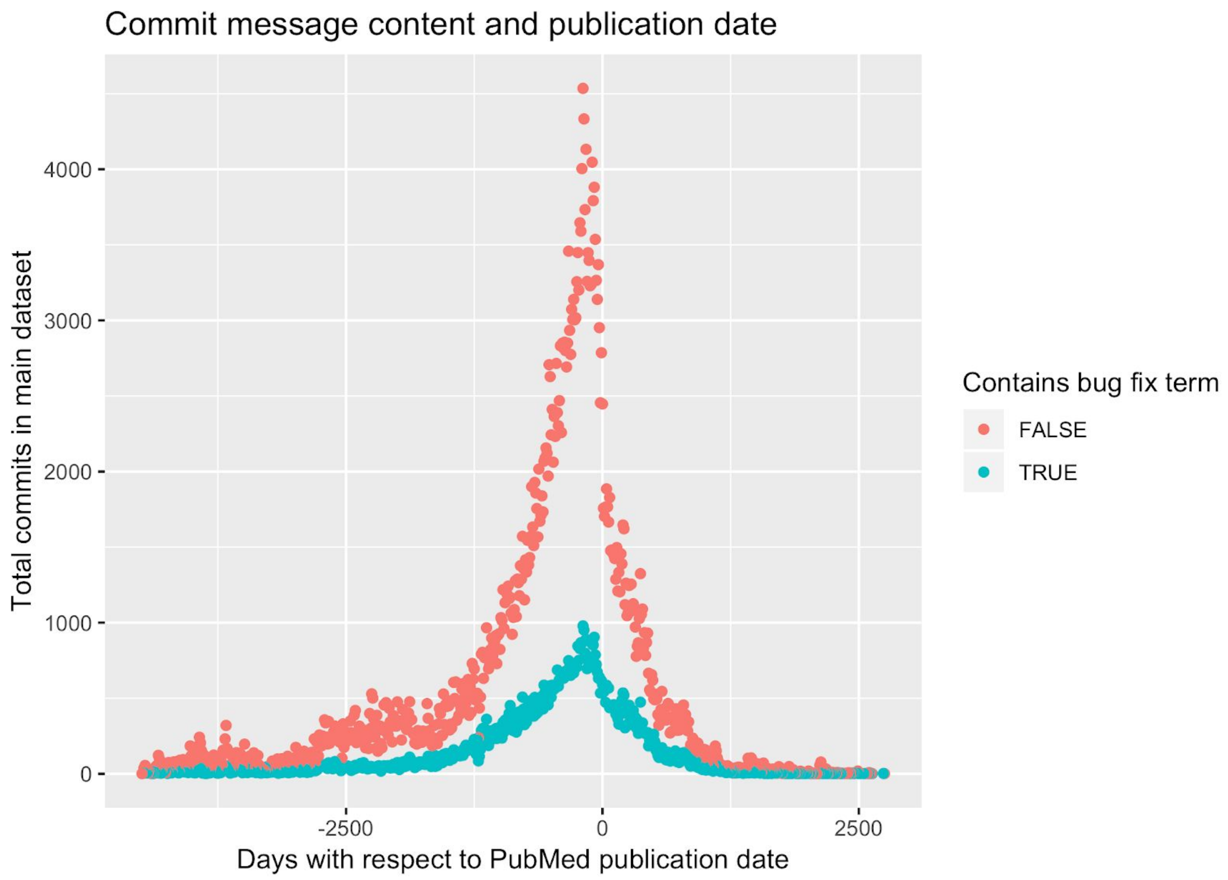

Fig K. Commit message content in the main dataset. We evaluate whether commit messages contain error-related keywords as defined in [25]. Commits are presented according to their relative timing with respect to the publication of the associated article (negative times are before article publication). Each dot represents all commits across the entire dataset for a 10-day interval with respect to the publication date. The figure shows an increase in overall commits approaching paper publication, but no disproportionate increase in bug fix commits as defined in [25].

References

1. bigrquery [Internet]. Github; Available: https://github.com/r-dbi/bigrquery
2. Scope Guidelines | Bioinformatics | Oxford Academic [Internet]. [cited 19 Mar 2018]. Available: https://academic.oup.com/bioinformatics/pages/scope_guidelines
3. Wikipedia contributors. Bioinformatics. In: Wikipedia, The Free Encyclopedia [Internet]. 9 Jun 2017 [cited 19 Mar 2018]. Available: https://en.wikipedia.org/w/index.php?title=Bioinformatics&oldid=784735926
4. pdfminer 20140328 : Python Package Index [Internet]. [cited 19 Mar 2018]. Available: https://pypi.python.org/pypi/pdfminer/
5. Prlić A, Yates A, Bliven SE, Rose PW, Jacobsen J, Troshin PV, et al. BioJava: an open-source framework for bioinformatics in 2012. Bioinformatics. 2012;28: 2693–2695. doi:10.1093/bioinformatics/bts494
6. BioJS [Internet]. [cited 19 Mar 2018]. Available: https://biojs.net/
7. Parnell LD, Lindenbaum P, Shameer K, Dall'Olio GM, Swan DC, Jensen LJ, et al. BioStar: an online question & answer resource for the bioinformatics community. PLoS Comput Biol. 2011;7: e1002216. doi:10.1371/journal.pcbi.1002216
8. Li H, Handsaker B, Wysoker A, Fennell T, Ruan J, Homer N, et al. The Sequence Alignment/Map format and SAMtools. Bioinformatics. 2009;25: 2078–2079. doi:10.1093/bioinformatics/btp352
9. Kovalchik S. RISmed: download content from NCBI databases. R package version. 2015;
10. GitHub API v3 | GitHub Developer Guide [Internet]. Github; Available: https://developer.github.com/v3/
11. Burnashev A. gspread [Internet]. Github; Available: https://github.com/burnash/gspread
12. Kjetil Jacobsen MFXJO. PycURL Home Page [Internet]. [cited 19 Mar 2018]. Available: http://pycurl.io/
13. Treat T. BigQuery-Python [Internet]. Github; Available: https://github.com/tylertreat/BigQuery-Python
14. Blei DM, Ng AY, Jordan MI. Latent Dirichlet Allocation. J Mach Learn Res. 2003;3: 993–1022. Available: http://www.jmlr.org/papers/volume3/blei03a/blei03a.pdf
15. Grün B, Hornik K. topicmodels: An R Package for Fitting Topic Models. Journal of Statistical Software, Articles. 2011;40: 1–30. doi:10.18637/jss.v040.i13
16. Silge J, Robinson D. Text Mining with R: A Tidy Approach [Internet]. "O'Reilly Media, Inc.";

2017. Available: https://market.android.com/details?id=book-qtcnDwAAQBAJ

17. cloc [Internet]. Github; Available: https://github.com/AlDanial/cloc

18. Wikipedia contributors. List of programming languages by type. In: Wikipedia, The Free Encyclopedia [Internet]. 12 Dec 2017 [cited 15 Mar 2018]. Available: https://en.wikipedia.org/w/index.php?title=List_of_programming_languages_by_type&oldid=814994307

19. Wikipedia contributors. Comparison of type systems. In: Wikipedia, The Free Encyclopedia [Internet]. 5 Sep 2017 [cited 15 Mar 2018]. Available: https://en.wikipedia.org/w/index.php?title=Comparison_of_type_systems&oldid=799049191

20. Strømgren C. Genderize.io | Determine the gender of a first name [Internet]. [cited 25 Jan 2018]. Available: https://genderize.io/

21. Shannon CE. A mathematical theory of communication. The Bell System Technical Journal. 1948;27: 379–423. doi:10.1002/j.1538-7305.1948.tb01338.x

22. Campbell K, Mínguez-Vera A. Gender Diversity in the Boardroom and Firm Financial Performance. J Bus Ethics. Springer Netherlands; 2008;83: 435–451. doi:10.1007/s10551-007-9630-y

23. Mínguez-Vera A, Martin A. Gender and management on Spanish SMEs: an empirical analysis. The International Journal of Human Resource Management. Routledge; 2011;22: 2852–2873. doi:10.1080/09585192.2011.599948

24. Boost C++ Libraries [Internet]. [cited 18 Mar 2018]. Available: http://www.boost.org/

25. Ray B, Posnett D, Filkov V, Devanbu P. A large scale study of programming languages and code quality in github. Proceedings of the 22nd ACM SIGSOFT International Symposium on Foundations of Software Engineering. ACM; 2014. pp. 155–165. doi:10.1145/2635868.2635922
